# Supplementary material for: Development and Pilot Use of a Questionnaire to Assess the Knowledge of Midwives and Pediatric Nurses on Maternal Use of Analgesics during Lactation
Source: Int J Environ Res Public Health. 2021 Nov 3;18(21):11555. doi: 10.3390/ijerph182111555 (PMC8583667; doi:10.3390/ijerph182111555)
Supplement: Supplementary file 1 [file ijerph-18-11555-s001.zip › supplement vragenlijst nederlandstalig.pdf]

Finale vragenlijst (Nederlandstalige versie, correcte antwoorden *aangeduid,* alle correcte antwoorden moeten aangeduid worden om te klasseren als een correct antwoord, aanpassingen op basis van de piloot test aangeduid als *geel+italics*)

Kennis van vroedvrouwen en pediatrisch verpleegkundigen over het gebruik van pijnstillers tijdens borstvoeding.

Risico's voor de zuigeling bij gebruik van pijnstillers tijdens de borstvoeding

V1 Ademhalingsdepressie: welke van de onderstaande geneesmiddelen kan mogelijk ademhalingsdepressie bij de zuigeling veroorzaken? *Duid aan (meerdere antwoorden mogelijk)*

Paracetamol

Ibuprofen

Aspirine/acetylsalicylzuur

Tramadol

Codeïne

Oxycodone

Ik weet het niet

V2 Bloedingen: welke van de onderstaande geneesmiddelen kan eventueel het risico op bloedingen bij de zuigeling verhogen? *Duid aan (meerdere antwoorden mogelijk)*

Paracetamol

Ibuprofen

Aspirine/acetylsalicylzuur

Tramadol

Codeïne

Oxycodone

Ik weet het niet

V3 Sufheid: welke van de onderstaande geneesmiddelen kan mogelijk sufheid bij de baby veroorzaken? *Duid aan (meerdere antwoorden mogelijk)*

Paracetamol

Ibuprofen

Aspirine/acetylsalicylzuur

Tramadol

Codeïne

Oxycodone

Ik weet het niet

Toeschietreflex

V4 Welke van onderstaande geneesmiddelen kunnen mogelijk de toeschietreflex bij de moeder verminderen? Duid aan (meerdere antwoorden mogelijk)

Paracetamol

Ibuprofen

Aspirine/acetylsalicylzuur

Tramadol

Codeïne

Oxycodone

Ik weet het niet

Veilig kortdurend en langdurig gebruik van pijnstillers tijdens de borstvoeding

V5 Paracetamol (500 - 1000 mg) mag tijdens de borstvoeding worden gebruikt gedurende 1 tot 3 dagen. *Duid één antwoord aan.*

Ja

Nee

Ik weet het niet

V6 Paracetamol (500 - 1000 mg) mag tijdens de borstvoeding worden gebruikt gedurende > 3 dagen. *Duid één antwoord aan.*

Ja

Nee

Ik weet het niet

V7 Ibuprofen (400 - 600 mg) mag tijdens de borstvoeding worden gebruikt gedurende 1 tot 3 dagen. *Duid één antwoord aan.*

Ja

Nee

Ik weet het niet

V8 Ibuprofen (400 - 600 mg) mag tijdens de borstvoeding worden gebruikt gedurende > 3 dagen. *Duid één antwoord aan.*

Ja

Nee

Ik weet het niet

V9 Aspirine (500 - 1000 mg) mag tijdens de borstvoeding worden gebruikt gedurende 1 tot 3 dagen. *Duid één antwoord aan.*

Ja

Nee

Ik weet het niet

V10 Aspirine (500 - 1000 mg) mag tijdens de borstvoeding worden gebruikt gedurende > 3 dagen. *Duid één antwoord aan.*

Ja

Nee

Ik weet het niet

V11 Ibuprofen heeft de voorkeur t.o.v. aspirine als pijnstiller wanneer paracetamol onvoldoende werkt. *Duid één antwoord aan.*

Ja

Nee

Ik weet het niet

V12 Tramadol (50 - 100 mg) mag tijdens de borstvoeding worden gebruikt gedurende 1 tot 3 dagen. *Duid één antwoord aan.*

Ja

Nee

Ik weet het niet

V13 Tramadol (50 - 100 mg) mag tijdens de borstvoeding worden gebruikt gedurende > 3 dagen. *Duid één antwoord aan.*

Ja

Nee

Ik weet het niet

V14 Paracetamol 500 mg + 30 mg codeïne mag tijdens de borstvoeding worden gebruikt gedurende 1 tot 3 dagen. *Duid één antwoord aan.*

Ja

Nee

Ik weet het niet

V15 Paracetamol 500 mg + 30 mg codeïne mag tijdens de borstvoeding worden gebruikt gedurende > 3 dagen. *Duid één antwoord aan.*

Ja

Nee

Ik weet het niet

V16 Oxycodone (5 - 10 mg) mag tijdens de borstvoeding worden gebruikt gedurende 1 tot 3 dagen. *Duid één antwoord aan.*

Ja

Nee

Ik weet het niet

V17 Oxycodone (5 - 10 mg) mag tijdens de borstvoeding worden gebruikt gedurende > 3 dagen. *Duid één antwoord aan.*

Ja

Nee

Ik weet het niet

Toegang tot en gebruik van informatiebronnen

V18 Ik zoek online informatie over pijnstillergebruik tijdens de borstvoeding als ik iets niet weet? Zo ja, noteer een paar bronnen die je kent/gebruikt.

Ja, en noteer (welke bronnen)

Nee

V19 Ik zoek informatie op via protocollen van de dienst over pijnstillergebruik tijdens de borstvoeding als ik iets niet weet.

Ja

Nee

V20 Ik vraag een gynaecoloog om informatie over pijnstillergebruik tijdens de borstvoeding als ik iets niet weet.

Ja

Nee

V21 Ik vraag een pediater/neonatoloog om informatie over pijnstillergebruik tijdens de borstvoeding als ik iets niet weet.

Ja

Nee

V22 Ik vraag een apotheker om informatie over pijnstillergebruik tijdens de borstvoeding als ik iets niet weet.

Ja

Nee

V23 Ik vraag een collega van mijn eigen team om informatie over pijnstillergebruik tijdens de borstvoeding als ik iets niet weet.

Ja

Nee

V24 Ik vraag een lactatiekundige om informatie over pijnstillergebruik tijdens de borstvoeding als ik iets niet weet.

Ja

Nee

V25 Ik vraag een vroedvrouw (in geval van een pediatrisch verpleegkundige) of pediatrisch verpleegkundige (in geval van vroedvrouw) om informatie over pijnstillergebruik tijdens de borstvoeding.

Ja

Nee

Zelf-rapportage kennis

V26 Ik vind dat ik als zorgverlener voldoende kennis heb over het gebruik van niet-narcotische pijnstillers (paracetamol, ibuprofen en aspirine) tijdens de borstvoeding.

Helemaal eens

Eens

Neutraal

Oneens

Helemaal oneens

V27 Ik vind dat ik als zorgverlener voldoende kennis heb over het gebruik van narcotische pijnstillers (tramadol, codeïne, oxycodone) tijdens de borstvoeding.

Helemaal eens

Eens

Neutraal

Oneens

Helemaal oneens

V28 Ik voel mij als zorgverlener bekwaam genoeg om correct advies te verlenen aan moeders over het gebruik van niet-narcotische pijnstillers (paracetamol, ibuprofen en aspirine) tijdens de borstvoeding.

Helemaal eens

Eens

Neutraal

Oneens

Helemaal oneens

V29 Ik voel mij als zorgverlener bekwaam genoeg om correct advies te verlenen aan moeders over het gebruik van narcotische pijnstillers (tramadol, codeïne, oxycodone) tijdens de borstvoeding.

Helemaal eens

Eens

Neutraal

Oneens

Helemaal oneens

V30 Ik heb in mijn basisopleiding voldoende educatie gekregen over de mogelijke risico's omtrent het gebruik van niet-narcotische pijnstillers (paracetamol, ibuprofen en aspirine tijdens de borstvoeding.

Helemaal eens

Eens

Neutraal

Oneens

Helemaal oneens

V31 Ik heb in mijn basisopleiding voldoende educatie gekregen over de mogelijke risico's omtrent het gebruik van narcotische pijnstillers (tramadol, codeïne, oxycodone) tijdens de borstvoeding.

Helemaal eens

Eens

Neutraal

Oneens

Helemaal oneens

V32 Ik vind dat het geven van informatie omtrent pijnstillergebruik tijdens de borstvoeding de verantwoordelijkheid is van: *Duid aan (meerdere antwoorden mogelijk)*

Kinderarts/Neonatoloog

Gynecoloog

Apotheker

Vroedvrouw

Pediatrisch verpleegkundige

V33 Ik vind dat het hebben van voldoende kennis tijdens de borstvoeding toebehoort aan: *Duid aan (meerdere antwoorden mogelijk)*

Kinderarts/Neonatoloog

Gynecoloog

Apotheker

Vroedvrouw

Pediatrisch verpleegkundige

Casussen vroedvrouwen

Casus 1, vroedvrouwen

Een moeder heeft gisteren een sectio gehad en mag omwille van haar voorgeschiedenis met bariatrische chirurgie geen NSAID's nemen. Mevrouw krijgt momenteel paracetamol tegen de pijn maar dit lijkt niet voldoende te zijn om de pijn onder controle te houden. Mevrouw geeft aan dat ze in het verleden al tramadol heeft gebruikt en vraagt aan de vroedvrouw om tramadol 50 mg te mogen innemen samen met paracetamol. Een belangrijk detail is dat mevrouw borstvoeding geeft. **Mag mevrouw tramadol 50 mg (tot 3x/24u indien nodig) innemen voor kortdurend gebruik (max 3 dagen)?** *Duid één antwoord aan.*

Ja, tramadol komt slechts in heel kleine hoeveelheden in de moedermelk terecht en brengt geen risico's voor de zuigeling met zich mee bij kortdurend gebruik. (1)

Ja, het kortdurend gebruik van tramadol kan veilig worden gebruikt, maar moeder en baby dienen wel geobserveerd te worden op symptomen. (2)

Nee, het gebruik van tramadol kan ook bij kortdurend gebruik, in kleine hoeveelheden in de moedermelk terecht komen en kan niet veilig worden gebruikt bij de zuigeling. (3)

Ik weet het niet. (4)

Casus 2, vroedvrouwen

Een moeder is twee weken geleden bevallen van een dochter. Mevrouw geeft exclusief borstvoeding maar dit gaat al enkele dagen minder vlot. Sinds eergisteren heeft ze hevige pijn in haar borst. Haar borst ziet ook rood en is gezwollen en er komt een pusafscheiding uit haar tepel. De vroedvrouw komt vandaag op huisbezoek en stelt vast dat mevrouw een zeer pijnlijk borstabces heeft. Mevrouw heeft thuis nog paracetamol 500 mg + 30 mg codeïne liggen. Dit had haar huisarts in het verleden eens voorgeschreven voor een migraine opstoot. **Ze vraagt zich af of het kortdurend gebruik van deze medicatie (tot 4x/24u, gedurende maximum 3 dagen) toegelaten is tijdens het geven van borstvoeding?** *Duid één antwoord aan.*

Ja, dit is toegelaten. Het kortdurend gebruik van deze medicatie brengt geen risico's voor de zuigeling met zich mee. (1)

Ja, dit is toegelaten. Het kortdurend gebruik van deze medicatie heeft geen schadelijke gevolgen voor de baby maar moeder en baby dienen wel geobserveerd te worden tijdens het gebruik van deze medicatie op ademhalingsdepressie en sedatie. (2)

Nee, dit is niet toegelaten. Het gebruik van paracetamol met codeïne tijdens de borstvoeding kan risico's voor de zuigeling met zich meebrengen en is dus afgeraden. Ik raad mevrouw een combinatie van paracetamol en ibuprofen aan en verwijs haar door naar een arts. (3)

Ik weet het niet. (4)

Casus 3, vroedvrouwen

Een moeder heeft gisteren een secundaire sectio ondergaan omwille van foetaal lijden. Het was mentaal en fysiek een zware ingreep voor haar. Er wordt geprobeerd om de pijn onder controle te houden met paracetamol en ibuprofen. Mevrouw geeft aan dat de pijn onvoldoende onder controle is en vraagt of er geen mogelijkheden zijn voor extra pijnstilling. Een assistent op de afdeling materniteit stelt voor om oxycodone 5 mg, een zwaardere pijnstiller, te geven bovenop de combinatie paracetamol en ibuprofen. De vroedvrouwen waarschuwen hem/haar dat mevrouw borstvoeding geeft en vragen zich af of dit geen risico's voor de baby met zich meebrengt. **De assistent beweert dat het gebruik van oxycodone 5mg voor maximum 3 dagen na elkaar 3x/24u geen schadelijke effecten heeft voor de zuigeling. Klopt zijn/haar uitspraak?** *Duid één antwoord aan*

Ja, oxycodone 5 mg brengt bij kortdurend gebruik geen risico's voor de zuigeling met zich mee, wel moet men de toeschietreflex observeren van de moeder. (1)

Nee, oxycodone 5 mg kan ook bij kortdurend gebruik risico's voor de zuigeling met zich meebrengen en wordt altijd (*toegevoegd na pilot testing*) afgeraden om te gebruiken tijdens het geven van borstvoeding. (2)

Nee, oxycodone 5 mg kan ook bij kortdurend gebruik risico's voor de zuigeling met zich meebrengen maar kan indien nodig (toegevoegd na pilot testing) wel voor kortdurend gebruik toegediend worden mits strikt (toegevoegd na pilot testing) observeren van moeder en zuigeling op sedatie en ademhalingsdepressie, alsook de toeschietreflex. (3)

Ik weet het niet. (4)

Casus 4, vroedvrouwen

Op de materniteit wordt een moeder heropgenomen omwille van mastitis. Ze geeft borstvoeding aan haar baby van 1 week oud. Ze nam twee uur geleden thuis een gram paracetamol in maar de pijn is nog steeds moeilijk uit te staan. Aangezien het te vroeg is om een nieuwe paracetamol te nemen, vraagt ze jou of ze éénmalig een tablet aspirine (1000 mg) mag innemen, ze heeft deze bij zich. **Ze vraagt aan jou of het mogelijk is om deze medicatie in te nemen?** *Duid één antwoord aan*

Nee, de vrouw mag deze medicatie niet nemen. Aspirine geeft een verhoogde kans op bloedingen bij de zuigeling. Paracetamol is de enige veilige pijnstiller. Ik kan de vrouw niks meer bij geven. (1)

Ja, het eenmalig gebruik van aspirine 1000 mg is veilig voor de zuigeling. Maar ik raad mevrouw ibuprofen aan i.p.v. aspirine, aangezien dit een beter alternatief is wanneer paracetamol onvoldoende werkt. (2)

Nee, de vrouw mag deze medicatie niet innemen. Aspirine geeft een verhoogde kans op bloedingen bij de zuigeling. Ik bel naar de gynaecoloog om tramadol op te starten. (3)

Ik weet het niet. (4)

<u>Casus 5, vroedvrouwen</u>

Mevrouw is twee dagen geleden bevallen van een zoon. Tijdens de kennismaking weet de vrouw je te vertellen dat ze tijdens de laatste weken van haar zwangerschap heel wat pijn aan haar rug had, ze wilde toen graag ibuprofen 600 mg innemen (3x/24u), maar dit mocht niet tijdens de zwangerschap. **Met paracetamol alleen is de pijn niet onder controle, mag mevrouw ibuprofen 600 mg innemen tijdens het geven van borstvoeding?** *Duid één antwoord aan.*

> ***<u>Ja, mevrouw mag 3x/24 u ibuprofen 600 mg nemen tijdens de borstvoeding. (1)</u>***
>
> Ja, mevrouw mag ibuprofen 600 mg nemen tijdens de borstvoeding, maar slechts 1x/24u. (2)
>
> Nee, het nemen van ibuprofen 600 mg raden we liever af tijdens het geven van borstvoeding. (3)
>
> Ik weet het niet. (4)

Casussen pediatrisch verpleegkundigen

Casus 1, pediatrisch verpleegkundigen

Een moeder is recent geopereerd aan een hernia. De chirurg schreef haar tramadol 50 mg, 3x/24u voor omdat paracetamol en ibuprofen onvoldoende hielpen. Ze mag dit maximum 5 dagen nemen, maar geeft exclusief borstvoeding aan haar 5 maanden oude zuigeling die bij u op pediatrie is opgenomen. **Mag mevrouw tramadol 50 mg (3x/24u) innemen voor 5 dagen?** *Duid één antwoord aan.*

Ja, tramadol komt slechts in heel kleine hoeveelheden in de moedermelk en is veilig te gebruiken gedurende 5 dagen. (1)

Ja, tramadol kan gedurende 5 dagen gebruikt worden, maar ik moet moeder en kind observeren op symptomen. (2)

Nee, het is niet aangeraden om tramadol gedurende 5 dagen te gebruiken in combinatie met het geven van borstvoeding. (3)

Ik weet het niet. (4)

Casus 2, pediatrisch verpleegkundigen

Een overigens gezonde a terme zuigeling van 1 maand oud is opgenomen met pyelonefritis op uw pediatrie afdeling. De moeder geeft exclusief borstvoeding, maar heeft beginnende mastitisklachten (roodheid, harde schijf in de borst, pijn). Paracetamol helpt niet voldoende om de pijn te onderdrukken. Ze wil de komende dagen ibuprofen 400 mg 3x/24u bijnemen. **Mag mevrouw dit nemen?** *Duid één antwoord aan.*

Ja, mevrouw mag 400 mg 3x/24 uur innemen. (1)

Nee, paracetamol is de enige veilige pijnstiller tijdens de borstvoeding. (2)

Nee, enkel bij kinderen ouder dan 3 maanden mag dit worden genomen. (3)

Ik weet het niet. (4)

Casus 3, pediatrisch verpleegkundigen

Een zuigeling van 4 maanden is opgenomen op uw afdeling pediatrie met een virale gastro-enteritis. Haar moeder heeft sinds gisteren hoofd- en nekpijn, vermoedelijk als gevolg van spanning door de opname van haar baby. Paracetamol biedt onvoldoende verlichting van de pijn. Vroeger nam ze gewoonlijk aspirine in tegen de hoofdpijn, haar ouders doen dit ook al jaren. Ze geeft borstvoeding en vraagt aan jou of ze eenmalig een tablet aspirine (1000 mg) mag innemen. **Mag mevrouw dit innemen?** *Duid één antwoord aan.*

Nee, de vrouw mag deze medicatie niet nemen. Aspirine geeft een verhoogde kans op bloedingen bij de zuigeling. Paracetamol is de enige veilige pijnstiller. Ik kan de vrouw niks anders aanraden. (1)

Ja, het eenmalig gebruik van aspirine 1000 mg is veilig voor de zuigeling. Maar ik raad mevrouw ibuprofen aan, aangezien dit een veiliger alternatief is tijdens borstvoeding wanneer paracetamol onvoldoende werkt. (2)

Ja, het eenmalig gebruik van aspirine 1000 mg is veilig. (3)

Ik weet het niet. (4)

Casus 4, pediatrisch verpleegkundigen

Een moeder is vorige week gaan tennissen en heeft hierbij een spierscheur in haar tussenribspier opgelopen. Paracetamol en ibuprofen helpen onvoldoende, ze blijft pijn hebben. Ze gaat naar de huisarts en hij schrijft haar tramadol 50 mg voor in te nemen indien de pijn niet meer te verdragen is. Mevrouw is terughoudend omtrent deze medicatie omdat ze exclusief borstvoeding geeft aan haar 3 maanden oude zuigeling die bij u op pediatrie is opgenomen. Ze vraagt op de afdeling pediatrie of het toegestaan is om deze medicatie te nemen. **Mag mevrouw dit innemen?** *Duid één antwoord aan.*

Ja, tramadol kan gebruikt worden, maar liefst beperken tot 3 dagen en mits observatie van moeder en zuigeling. (1)

Ja, tramadol kan gebruikt worden, (mits observatie van moeder en zuigeling = *verwijderd na pilot testing*), zowel voor langdurig als kortdurend gebruik. (2)

Nee, tramadol is niet veilig te gebruiken bij de zuigeling, ook niet bij kortdurend gebruik. (3)

Ik weet het niet. (4)

Casus 5, pediatrisch verpleegkundigen

Het is winter en de pediatrie afdeling ligt vol met RSV infecties. Gust, een zuigeling van 4 maanden ligt al even in het ziekenhuis. Mevrouw ervaart de laatste dagen veel stress en krijgt een migraine aanval. De huisdokter had voor haar zwangerschap al eens **paracetamol 500 mg + 30 mg codeïne** voorgeschreven en ze heeft hiervan nog een half doosje thuis liggen. Ze vraagt aan jou of ze dit mag nemen, tot 4x/24u, zolang het nodig is. Gust krijgt afgekolfde moedermelk, die omwille van de huidige RSV infectie per sonde wordt gegeven. **Mag mevrouw dit innemen?** *Duid één antwoord aan.*

Ja, het nemen van codeïne tijdens het geven van borstvoeding brengt geen risico's voor de zuigeling met zich mee op. Maar paracetamol en ibuprofen worden als eerste keuze preparaten aangeraden. (1)

Neen, codeïne tijdens het geven van borstvoeding is afgeraden. Paracetamol en ibuprofen worden als eerste keuze preparaten aangeraden. (2)

Ja, codeïne kan worden gebruikt, maar enkel voor kortdurend gebruik (2-3 dagen). Maar paracetamol en ibuprofen worden als eerste keuze preparaten aangeraden. (3)

Ik weet het niet. (4)
