# Supplementary material for: Development and Pilot Use of a Questionnaire to Assess the Knowledge of Midwives and Pediatric Nurses on Maternal Use of Analgesics during Lactation
Source: Int J Environ Res Public Health. 2021 Nov 3;18(21):11555. doi: 10.3390/ijerph182111555 (PMC8583667; doi:10.3390/ijerph182111555)
Supplement: Supplementary file 1 [file ijerph-18-11555-s001.zip › supplement, Final questionnaire 2.0.pdf]

Final questionnaire (English version, correct answers *highlighted*, all correct answers should be provided to classify as correct response, adaptations based on the pilot testing are indicated in *yellow+italics*)

Knowledge of midwives and pediatric nurses on the use of analgesics during lactation

Risks for the infant during the maternal use of analgesics during lactation

Q1 Respiratory depression: which of the medicines listed below may cause respiratory depression in the nursing infant? *(more than 1 answer can be correct)*

Acetaminophen/paracetamol

Ibuprofen

Aspirin/acetylsalicylic acid

Tramadol

Codeine

Oxycodone

I don't know

Q2 Bleeding: which of the medicines listed below may increase the risk of bleeding in the nursing infant? *(more than 1 answer can be correct)*

Acetaminophen/paracetamol

Ibuprofen

Aspirin/acetylsalicylic acid

Tramadol

Codeine

Oxycodone

I don't know

Q3 Sleepiness/sedation: which of the medicines listed below may increase the risk of bleeding in the nursing infant? *(more than 1 answer can be correct)*

Acetaminophen/paracetamol

Ibuprofen

Aspirin/acetylsalicylic acid

Tramadol

Codeine

Oxycodone

I don’t know

Let-down reflexes

Q4 Which of the medicines listed below may lower the let-down reflex in the mother? *(more than 1 answer can be correct)*

Acetaminophen/paracetamol

Ibuprofen

Aspirin/acetylsalicylic acid

Tramadol

Codeine

Oxycodone

I don’t know

Safe short and prolonged use of analgesics during lactation

Q5 Acetaminophen (paracetamol)(500-1000 mg doses) can be used safely during lactation for 1-3 days. *(only provide one answer)*

Yes

No

I don’t know

Q6 Acetaminophen (paracetamol)(500-1000 mg doses) can be used safely during lactation for > 3 days. *(only provide one answer)*

Yes

No

I don’t know

Q7 Ibuprofen (400-600 mg doses) can be used safely during lactation for 1-3 days. *(only provide one answer)*

Yes

No

I don’t know

Q8 Ibuprofen (400-600 mg doses) can be used safely during lactation for > 3 days. *(only provide one answer)*

Yes

No

I don’t know

Q9 Aspirin (acetylsalicylic acid, 500-1000 mg doses) can be used safely during lactation for 1-3 days. *(only provide one answer)*

<u>Yes</u>

No

I don’t know

Q10 Aspirin (acetylsalicylic acid, 500-1000 mg doses) can be used safely during lactation for > 3 days. *(only provide one answer)*

Yes

<u>No</u>

I don’t know

Q11 Ibuprofen is the preferred option compared to aspirin (acetylsalicyl acid) as analgesic if acetaminophen (paracetamol) is not sufficiently effective. *(only provide one answer)*

<u>Yes</u>

No

I don’t know

Q12 Tramadol (50-100 mg doses) can be used safely during lactation for 1-3 days. *(only provide one answer)*

<u>Yes</u>

No

I don’t know

Q13 Tramadol (50-100 mg doses) can be used safely during lactation for > 3 days. *(only provide one answer)*

<u>Yes</u>

No

I don’t know

Q14 Acetaminophen (paracetamol) 500 mg + 30 mg codeine can be used safely during lactation for 1-3 days. *(only provide one answer)*

<u>Yes</u>

No

I don’t know

Q15 Acetaminophen (paracetamol) 500 mg + 30 mg codeine can be used safely during lactation for > 3 days. *(only provide one answer)*

Yes

No

I don't know

Q16 Oxycodone (5-10 mg) can be used safely during lactation for 1-3 days. *(only provide one answer)*

<u>Yes</u>

No

I don't know

Q17 Oxycodone (5-10 mg) can be used safely during lactation for > 3 days. *(only provide one answer)*

Yes

<u>No</u>

I don't know

<u>Access to and use of sources of information</u>

Q18 I search for online information on the use of analgesics during lactation if I don't know something. If so, please mention some sources you know/use.

Yes, (and add those sources)

No

Q19 I search for protocols of the department on the use of analgesics during lactation if I don't know something.

Yes

No

Q20 I ask a gynecologist for information on the use of analgesics during lactation if I don't know something.

Yes

No

Q21 I ask a pediatrician/neonatologist for information on the use of analgesics during lactation if I don't know something.

Yes

No

Q22 I ask a pharmacist for information on the use of analgesics during lactation if I don’t know something.

Yes

No

Q23 I ask a colleague of my own team for information on the use of analgesics during lactation if I don’t know something.

Yes

No

Q24 I ask a lactation specialist for information on the use of analgesics during lactation if I don’t know something.

Yes

No

Q25 I ask a midwife (in the event of a pediatric nurse) or a pediatric nurse (in the event of a midwife) for information on the use of analgesics during lactation if I don’t know something.

Yes

No

Self-reported knowledge

Q26 As healthcare provider, I have sufficient knowledge of the usage of non-narcotic analgesics (acetaminophen/paracetamol, ibuprofen and aspirin/acetylsalicylic acid) during lactation.

Fully agree

Agree

Neutral

Disagree

Fully disagree

Q27 As healthcare provider, I have sufficient knowledge of the usage of narcotic analgesics (tramadol, codeine, oxycodone) during lactation.

Fully agree

Agree

Neutral

Disagree

Fully disagree

Q28 As healthcare provider, I am sufficiently competent to provide correct advice to women on the usage of non-narcotic analgesics (acetaminophen/paracetamol, ibuprofen and aspirin/acetylsalicylic acid) during lactation.

Fully agree

Agree

Neutral

Disagree

Fully disagree

Q29 As healthcare provider, I am sufficiently competent to provide correct advice to women on the usage of narcotic analgesics (tramadol, codeine, oxycodone) during lactation.

Fully agree

Agree

Neutral

Disagree

Fully disagree

Q30 I have received sufficient education on the potential risks on the use of non-narcotic analgesics (acetaminophen/paracetamol, ibuprofen and aspirin/acetylsalicylic acid) during lactation.

Fully agree

Agree

Neutral

Disagree

Fully disagree

Q31 I have received sufficient education on the potential risks on the use of narcotic analgesics (tramadol, codeine, oxycodone) during lactation.

Fully agree

Agree

Neutral

Disagree

Fully disagree

Q32 In my opinion, providing information on the use of analgesics during lactation is the responsibility of the: (*more than 1 answer can be provided*)

Pediatrician/Neonatologist

Gynecologist

Pharmacist

Midwife

Pediatric nurse

Q33 In my opinion, having sufficient knowledge on the use of analgesics during lactation is expected from a: (*more than 1 answer can be provided*)

Pediatrician/Neonatologist

Gynecologist

Pharmacist

Midwife

Pediatric nurse

Clinical cases, midwives

Case 1, midwives

A mother underwent a caesarean delivery yesterday, and cannot be treated with non-steroidal anti-inflammatory drugs due to a history of bariatric surgery. The woman is currently being treated with acetaminophen/paracetamol, but this is not sufficiently effective. The woman indicates that she has used tramadol previously, and asks the midwife to add tramadol 50 mg to the current treatment with acetaminophen/paracetamol. A relevant detail is that the mother is currently breastfeeding. **Can she take tramadol 50 mg (as needed, up to 3x/24h) for a short period of time (max 3 days) ?** *(indicate one answer)*

Yes, tramadol only appears in very limited amounts in human milk and does not result in any risk for the lactating newborn when used for a short period of time. (1)

Yes, the short-time intake of tramadol can be used safely, but mother and newborn should be observed for symptoms. (2)

No, tramadol may – also during short time use – appear in limited amounts in human milk and cannot be used safely for the lactating newborn. (3)

I don't know. (4)

Case 2, midwives

A mother delivered a girl 2 weeks ago. The mother is giving exclusive breastfeeding, but this became more difficult in the last days. Since the day before yesterday, she has severe pain in her breast. This breast also appeared to be red and swollen, with purulent discharge. The midwife visited the patient at home and diagnosed a painful breast abscess. The mother still has acetaminophen/paracetamol 500 mg + codeine 30 mg tablets at home. Those have been prescribed in the past by the general practitioner to treat migraine attacks. **The woman wonders if short time use of this medicine (up to 4 tablets/day, for maximal 3 days) is allowed when breastfeeding ?** *(indicate one answer)*

Yes, this is allowed, as short time use of this medicine is not associated with risks for the lactating infant. (1)

Yes, this is allowed. Short time use of this medicine has no harmful consequences for the lactating infant, but mother and infant should be observed during the use of this medicine for respiratory depression and sedation. (2)

No, this is not allowed. The use of acetaminophen/paracetamol+codeine while breastfeeding may result in risks for the nursing infant and should be discouraged. I suggest combining acetaminophen/paracetamol with ibuprofen, and refer her to a physician. (3)

I don't know. (4)

Case 3, midwives

A mother underwent yesterday a secondary (emergency) caesarean section because of fetal distress. This was for her a major intervention, both mentally and physically. One tries to control her pain with acetaminophen/paracetamol and ibuprofen. The mother indicates that the pain is insufficiently controlled, and requests additional analgesia. A trainee at the maternity ward suggests using oxycodone 5 mg, in combination with acetaminophen/ paracetamol and ibuprofen. The midwives mention that the mother is breastfeeding, and wonder if this may not result in risks for the newborn. **The trainee states that the use of oxycodone 5 mg for maximal three consecutive days (3x/24 h) does not result in harmful effects for the newborn. Is this correct?** *(indicate one answer)*

Yes, oxycodone 5 mg - for short time use - will not result in risks for the newborn, but the maternal led-down reflex should be observed. (1)

No, oxycodone 5 mg - also for short time use - may result in risks for the newborn, and is always (*added after pilot testing*) discouraged during lactation.(2)

No, oxycodone 5 mg - also for short time use - may result in risks for the newborn, but can be used if indicated (added after pilot testing) for a short time if the mother and the newborn are strickly (added after pilot testing) monitored for sedation, respiratory depression, and reduced (maternal) led-down reflex. (3)

I don’t know. (4)

Case 4, midwives

A mother is readmitted on the maternity ward because of mastitis. The mother is currently breastfeeding her newborn of 1 week old. About 2 hours ago, the woman took acetaminophen/paracetamol 1 g at home, but the pain is still difficult to sustain. As it is too soon to take another acetaminophen/paracetamol tablet, she wonders if she can take 1000 mg aspirin (acetylsalicylic acid) in addition, as she has this with her. **She asks you if she can take this medicine?** *(indicate one answer)*

No, she is not allowed to take this medicine, as aspirin/acetylsalicylic acid is associated with a higher risk for bleeding in the newborn. Acetaminophen/paracetamol is the only safe analgesic. I cannot give the woman any other medicine. (1)

Yes, the single use of aspirin/acetylsalicylic acid is safe for the newborn. However, I recommend ibuprofen instead of aspirin/acetylsalicylic acid, as this is a better alternative in the event of insufficient effect of acetaminophen/paracetamol. (2)

No, she is not allowed to take this medicine as aspirin/acetylsalicylic acid is associated with a higher risk for bleeding in the newborn. I call the gynecologist to prescribe tramadol. (3)

I don’t know. (4)

Case 5, midwives

A women gave birth to a son. During your intake on the ward, she tells you that she suffered quite significantly from back pain in the last weeks of her pregnancy. A that time, she wanted to take ibuprofen 600 mg (3x/24h), but this was not allowed during pregnancy. **However, the pain is not under control with only acetaminophen/paracetamol. Can she take ibuprofen (600 mg) while breastfeeding ?** *(indicate one answer)*

Yes, she can take 3x/24 h, ibuprofen 600 mg during lactation. (1)

Yes, she can take ibuprofen 600 mg during lactation, but only once daily (1x/24h). (2)

No, the intake of ibuprofen 600 mg during lactation is discouraged. (3)

I don’t know. (4)

<u>Clinical cases, pediatric nurses</u>

<u>Case 1, pediatric nurses</u>

A mother recently underwent surgery (hernia repair). The surgeon prescribed tramadol, 50 mg, 3x/24h as acetaminophen/paracetamol and ibuprofen were not sufficiently effective. She is allowed to take this drug for 5 days, but is also breastfeeding her 5 months old infant, who is just admitted on the pediatric unit. **Is this woman allowed to take tramadol 50 mg (3x/24h) for 5 days?** *(indicate one answer)*

Yes, tramadol only appears in very limited amount in the human milk, and is safe to be used for 5 days. (1)

<u>Yes, tramadol can be used for 5 days, but I have to monitor mother and infant for any symptoms. (2)</u>

No, it is not recommended to combine the use of tramadol for 5 days with breastfeeding. (3)

I don’t know. (4)

<u>Case 2, pediatric nurses</u>

An otherwise healthy term newborn of 1 month old is admitted with pyelonephritis on your pediatric ward. The mother gives exclusive breastfeeding, but has preliminary symptoms of mastitis (redness, tenderness, pain). Acetaminophen/paracetamol is not sufficient to control the pain. She would like to add ibuprofen 400 mg 3x/24h to this treatment regimen. **Can she take this?** *(indicate one answer)*

<u>Yes, she can take ibuprofen 400 mg, 3x/24 h. (1)</u>

No, acetaminophen/paracetamol is the only safe analgesic during lactation. (2)

No, ibuprofen can only be taken by breastfeeding women in the event that nursing infants are older than 3 months. (3)

I don’t know. (4)

Case 3, pediatric nurses

An infant of 4 months has been admitted on your pediatric ward because of viral gastro-enteritis. Her mother has head and neck pain, likely stress related due to the admission of her infant. Acetaminophen/paracetamol is not sufficient to relieve the pain. The woman usually takes aspirin/acetylsalicylic acid for headaches, similar to what her parents already do for years. As the woman is currently breastfeeding, she asks you if she can take a single aspirin/acetylsalicylic acid tablet of 1000 mg? **Can she take this ?** *(indicate one answer)*

No, she is not allowed to take this medicine, as aspirin/acetylsalicylic acid is associated with a higher risk for bleeding in the newborn. Acetaminophen/paracetamol is the only safe analgesic. I cannot give the woman any other medicine. (1)

Yes, the single use of aspirin/acetylsalicylic acid is safe for the newborn. However, I recommend ibuprofen instead of aspirin/acetylsalicylic acid, as this is a safer alternative during breastfeeding in the event of insufficient effect of acetaminophen/paracetamol. (2)

Yes, the single tablet of 1000 mg aspirin/acetylsalicylic acid can be used safely. (3)

I don't know. (4)

Case 4, pediatric nurses

A mother got an intercostal muscle tear while playing tennis last week. Acetaminophen/paracetamol and ibuprofen are not sufficiently effective, as she still is in pain. After a consult of the general practitioner, tramadol 50 mg has been prescribed as add on (in the event of unsustainable pain). The mother is hesitant to take this drug as she gives exclusive breastfeeding to her 3 month old infant, who is admitted on the pediatric ward. On this ward, she asks if she is allowed to take this medicine. **Can she take this?** *(indicate one answer)*

Yes, tramadol can be used, but it is preferred to restrict its usage to 3 days, and mother and infant should be observed. (1)

Yes, tramadol can be used (if the mother and infant are observed = *removed after pilot testing*), irrespective of short or prolonged use. (2)

No, tramadol is not safe for the nursing infant, neither for short term use. (3)

I don't know. (4)

Case 5, pediatric nurses

It is winter, and the pediatric ward is full of patients with RSV infections. Gust, an infant of 4 months has already been admitted for some time. His mother is stressed over the last days, and has a migraine attack. Before her pregnancy, the general practitioner had already prescribed her **acetaminophen/paracetamol 500 mg + codeine 30 mg**, of which she still has a half a box at home. She asks you if she can take this, up to 4x/24 h, as long as needed. Gust currently receives expressed human milk, administered by naso-gastric tube because of the ongoing RSV infection. **Can she take this medicine?** *(indicate one answer)*

Yes, codeine use during lactation does not result in risks for the infant. However, acetaminophen/paracetamol and ibuprofen are recommended as first line medicines. (1)

No, codeine use while breastfeeding is not recommended. Acetaminophen/paracetamol and ibuprofen are recommended as first line medicines. (2)

Yes, codeine can be used during lactation, but only for short term use (2-3 days). However, acetaminophen/paracetamol and ibuprofen are recommended as first line medicines. (3)

I don’t know. (4)
